# Stereotactic Radiofrequency Ablation of Liver Tumors in Octogenarians

**DOI:** 10.3389/fonc.2019.00929

**Published:** 2019-09-19

**Authors:** Peter Schullian, Daniel Putzer, Michael A. Silva, Gregor Laimer, Christian Kolbitsch, Reto Bale

**Affiliations:** ^1^Section of Interventional Oncology - Microinvasive Therapy, Department of Radiology, Medical University of Innsbruck, Innsbruck, Austria; ^2^Department of Hepatobiliary and Pancreatic Surgery, Oxford University Hospitals NHS Foundation Trust, Oxford, United Kingdom; ^3^Department of Anesthesia, Medical University of Innsbruck, Innsbruck, Austria

**Keywords:** radiofrequency ablation, octogenarian, liver, tumor, stereotaxy

## Abstract

**Purpose:** This study aimed to evaluate the efficacy and overall clinical outcome of patients over the age of 80 undergoing stereotactic radiofrequency ablation (SRFA) and to compare the results to a younger population with propensity score matching.

**Materials and Methods:** Between 2006 and 2018 36 patients aged between 80 and 90 years underwent 46 SRFA sessions of 70 primary and secondary liver tumors. For comparison of treatment safety and efficacy 36 younger patients were selected with propensity score matching by the R package “MatchIt” in this retrospective, single-center study.

**Results:** 68/70 tumors were successfully ablated at first ablation session (97% primary technical efficacy rate). Local tumor recurrence developed in 5 of 70 nodules (7.1%). The complication rate above Clavien-Dindo Grade III was 6.5% (3 of 46). The overall survival (OS) rates at 1-, 3-, and 5- years from the date of the first SRFA were 84.6, 50.5, and 37.9% for HCC patients and 87.5%, 52.5% at 1-, and 3-years for CRC patients. The disease-free survival (DFS) for HCC patients after SRFA was 79.1, 35.6, and 23.7%, at 1-, 3-, and 5- years, and 75%, 22.5% at 1-, and 3-years for CRC patients. There were no significant differences in terms of technical efficacy, local recurrences, major complications, OS and DFS compared to the control group.

**Conclusion:** SRFA in octogenarians is a safe, feasible and useful option in the management of primary or metastatic liver tumors with no significant difference in outcomes compared to a younger control group.

## Introduction

Worldwide the population is aging (1) due to rising living standards and life expectancy. In Western Europe, life expectancy has increased from 65 years for males and 70 years for females in 1950 to 79 years and 84 years in 2017, respectively (2). Moreover, life expectancy in Western Europe is expected to further rise to 90 years for males and 94 years for females in the year 2100 (2). In Austria, the number of people older than 80 years increased from 280,000 (3.6% of the population) in 2000 to 450,000 (5.4% of the population) in 2017 (3). With this trend, the incidence of co-morbidities and malignancies also rise in a larger cohort of patients. Hospitalization of older patients is commonly followed by an irreversible decline in functional status, i.e., losing independence in basic activities of daily living. This loss of independence is not only a result of the acute condition that lead to admission, but also substantially related to the duration and mode of management (4–6). Postoperative delirium occurs in 15–53% (7) of older patients after surgical treatment and its incidence can reach 70–87% (8) among patients admitted to postoperative intensive care unit.

The liver is a common site for malignant neoplasms. Where applicable, hepatic resection (HR) is still considered the gold standard in the management of primary or secondary liver tumors. However, HR is associated with mortality and morbidity rates of 5% and up to 40%, respectively, in older patients (9–11). Addressing expectations of rapid post-procedure recovery with short hospitalization, radiofrequency (RF) ablation with its minimal invasive nature has been increasingly accepted as an alternative in the management of primary or metastatic liver tumors (12, 13). This is more so though not exclusive in older patients. Other than for a few studies however such as Kaibori et al. (14), there is a paucity of data on the short and long term outcomes following RF ablation of liver tumors in the older.

This study aimed at evaluating the technical efficacy and overall clinical outcome of patients over the age of 80 undergoing SRFA for liver tumors and to compare the results to a younger population with propensity score matching.

## Materials and Methods

### Patient Cohort and Inclusion Criteria

The Institutional Review Board approved this study. All patients included the study had granted informed consent for their data to be used. In all patients, the treatment plan was established by a multidisciplinary tumor board consisting of hepatologists, oncologists, transplant and liver surgeons, and interventional radiologists. Decisions for therapy were based on tumor characteristics, liver function, anatomical considerations, and performance status. Age alone was not an exclusion criterion. Due to the multiprobe approach there were no limitations in the size or number of tumors.

**Figure 1:**
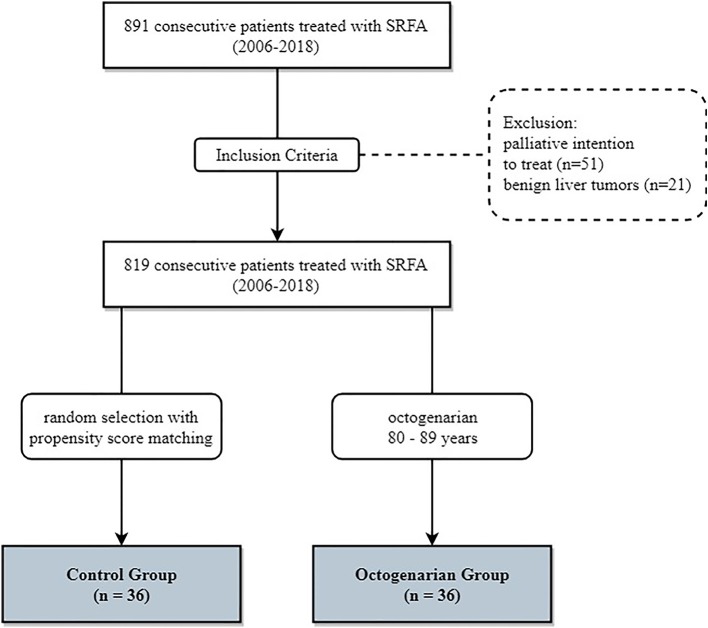
Flowchart of group assignment.

Data was reviewed retrospectively in a single-center, single-arm study. Between 2006 and 2018 891 consecutive patients were treated by SRFA. Seventy-two patients with invasion of the portal vein or extended tumor spread with subsequent palliative intention to treat or benign liver tumors, were excluded (Figure 1). Thirty-six patients over 80 years of age at treatment were included. For comparison 36 younger patients (median age 62 years, ranging from 38 to 79) were selected using nearest neighbor propensity score matching by the R package “MatchIt” with sex, tumor type, number and size and liver function as matching variables. The baseline characteristics of the two groups are shown in Table 1.

**Table 1:** Patient characteristics of 36 patients undergoing 46 SRFA sessions of 70 nodules in the octogenarian group and of 36 patients undergoing 67 SRFA sessions of 113 nodules in the control group.

**Patient characteristics**	**Octogenarian Gr**.	**Control Gr**.
Age, years (range)	81.5 (80–88)	61.5 (38–79)
Sex (female/male), *n* (%)	12/24 (33.3/66.7)	13/23 (36.1/63.9)
Tumor type, *n* (%)
HCC, *n* (%)	18 (50.0)	16 (44.4)
ICC, *n* (%)	3 (8.3)	3 (8.3)
Metastasis, *n* (%)	15 (41.7)	17 (47.2)
Colorectal, *n* (%)	9 (60.0)	9 (60.0)
Other, *n* (%)	6 (40.0)	6 (40.0)
Concomitant disease, *n* (%)	34 (94.4)	22 (61.1)
Cardiovascular, *n* (%)	25 (69.4)	15 (41.7)
Hypertension, *n* (%)	20	12
Hypercholesterolemia, *n*	6	3
Coronary artery disease, *n*	8	5
Cardiac insufficiency, *n*	5	2
Cerebral insult	5	3
Respiratory, *n* (%)	3 (8.3)	–
COPD, *n* (%)	3	–
Asthma, *n* (%)	–	–
Metabolic, *n* (%)	17 (47.2)	6 (16.7)
Diabetes, *n*	12	4
Thyroid disease, *n*	6	4
Cirrhosis, *n* (%)	12 (33.3)	12 (33.3)
Child A, *n* (%)	10 (91.7)	10 (91.7)
Child B, *n* (%)	2 (8.3)	2 (8.3)
Child C, *n* (%)	–	–
Renal failure, *n* (%)	7 (19.4)	2 (5.5)
Secondary tumor, *n* (%)	6 (16.7)	3 (8.3)
Charlson comorbidity index, median (range)	9.5 (6–16)	6 (2–13)
Tumor size, median	2.7 cm (1.5–9 cm)	2.5 cm (1.5–11 cm)
Tumor number at begin, median (range)	1 (1–5)	1 (1–4)
Total treated tumors, median (range)	1.5 (1–5)	2 (1–14)
Ablations per patient, median (range)	1 (1–3)	1 (1–5)

*SRFA, stereotactic radiofrequency ablation; HCC, hepatocellular carcinoma; ICC, intrahepatic cholangiocarcinoma; Child, Child Pugh Score; Gr., group*.

A platelet count of <50,000/mm^3^ and prothrombin activity <50% were exclusion criteria for RF ablation. At least two dynamic imaging modalities (CT or MRI) were used in initial staging. All tumors underwent biopsy to confirm diagnosis at the time of the RF ablation.

### Multi-Probe Stereotactic Radiofrequency Ablation

**Figure 2:**
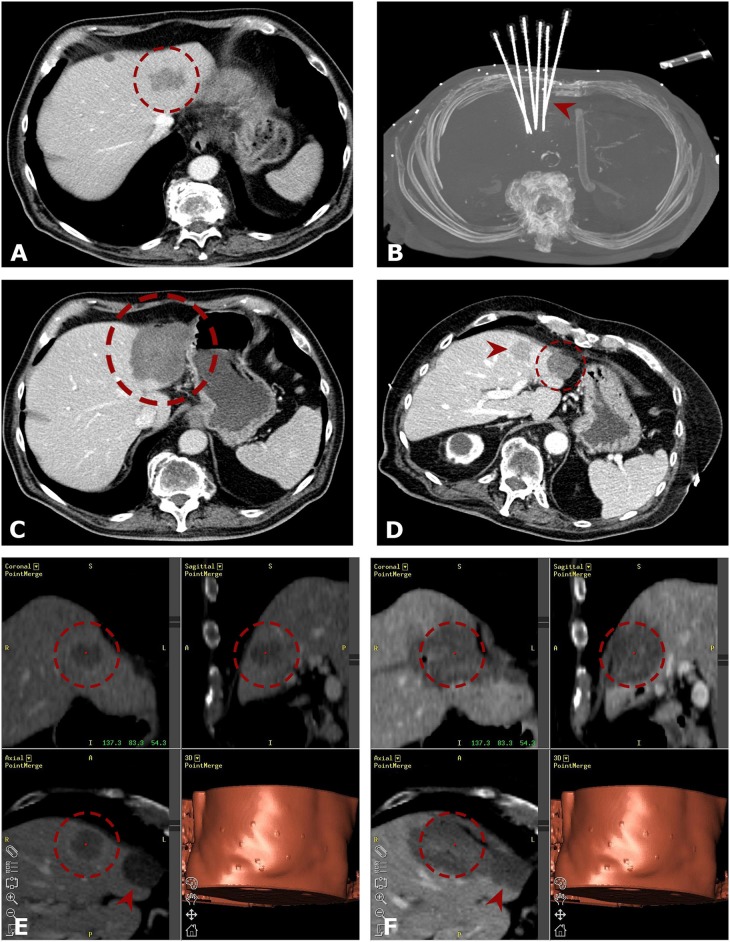
Case of a 80-years old male with a singular 4.0 cm HCC in segment II. **(A)** Portal venous phase initial CT-scan with a hypo-enhancing nodule in segment II (*red dashed circle*). The enhancement of the HCC is partly atypical (slight arterial flushing). **(B)** MIP of the native control CT with 6 needles in place (*red arrowhead*). **(C)** Axial portal venous ce-enhanced CT after 3 months showing complete coagulation zone (*red dashed circle*). **(D)** The *red dashed circle* is marking the progressively shrinking coagulation zone at 48 months. New distant tumor recurrence is highlighted by the *red arrowhead*. **(E,F)** Repeated SRFA of the distant tumor recurrence (*red dashed circle*). Fused images from the navigation system with 3D views from planning CT **(E)** and final control CT **(F)** with complete necrosis including a sufficient ablation margin. The *red arrowhead* is marking the initial ablation zone.

**Figure 3:**
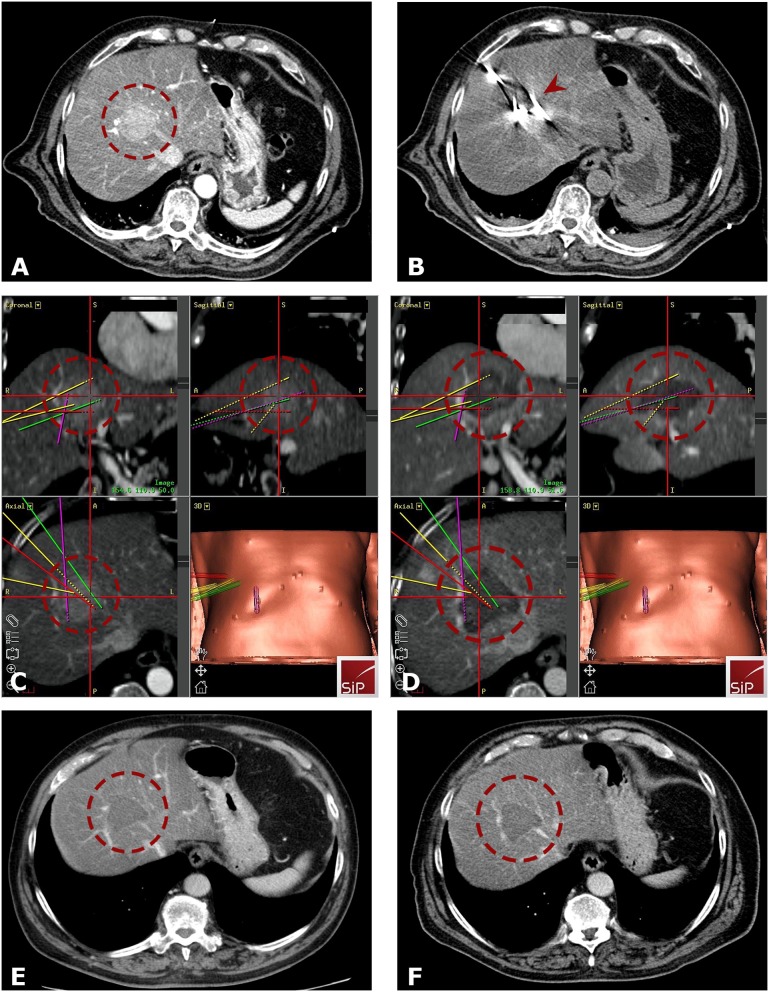
Case of a 81-years old male with a HCC in segment VIII. **(A)** Arterial phase planning CT with a 4.5 cm HCC close to portal vein and infiltration/obstruction of the middle hepatic vein (*red dashed circle*). **(B)** Native control CT showing in total 5 inserted coaxial needles (*red arrowhead*). **(C,D)** Fused images from the navigation system with 3D views from arterial phase planning CT **(A)** and final control CT **(D)** with complete necrosis including a sufficient ablation margin (*red dashed circle*). The colored lines correspond to the predefined paths. **(E,F)** Follow- up CT scans after 18 **(E)** and 24 months **(F)** with no evidence of local tumor recurrence (*red dashed circle* is marking the coagulation zone).

The technique of SRFA has been previously described (15, 16). Briefly, the whole procedure is carried out in the interventional CT-suite. The anesthetized patients were fixated on the CT-table by a single (Bluebag, Medical Intelligence Inc., Schwabmünchen, Germany) or double vacuum fixation technique (BodyFix, Medical Intelligence Inc., Schwabmünchen, Germany) with maximal muscle relaxation. Ten to fifteen registration markers were broadly attached to the skin of the thorax and upper abdomen for image-to-patient registration (Beekley Spots, Beekley Corporation, Bristol, CT, USA). Subsequently, a contrast-enhanced planning CT (Siemens SOMATOM Sensation Open, sliding gantry with 82 cm diameter, Siemens AG, Erlangen, Germany) with 3 mm slice thickness was performed. For compensation of the respiratory motion, temporary disconnections of the endotracheal tube (ETT) were performed during the planning CT, each stereotactic needle placement and the final control CT. The CT-data were transferred to the optical-based navigation system (Stealth Station Treon plus, Medtronic Inc., Louisville, KY, USA). With the navigation system one or multiple trajectories were planned according to the multiplanar and 3D reconstructed images. In order to achieve complete necrosis of the entire tumor tissue with an appropriate circumferential safety margin, RFA electrodes were aligned to each other with a maximum distance of 2 cm to each other. After registration, an accuracy check and sterile draping, the ATLAS aiming device (Medical Intelligence Inc., Schwabmünchen, Germany) was used for navigated trajectory alignment. One after another 15G/17.2 cm coaxial needles (Bard Inc., Covington, GA, USA) was advanced through the aiming device during temporary ETT disconnection without real-time imaging control. The navigation software automatically calculated the depth from the aiming device to the target. The coaxial needles served as guides for the radiofrequency electrodes. For verification of correct needle placement, a native control CT was performed in ETT disconnection and fused with the planning CT using the navigation system's image 3D registration algorithm. Through the coaxial needles, a 16G biopsy sample was obtained in all cases. After that, RF-electrodes (Cool-tip, Medtronic, Mansfield, MA, USA) were introduced through the coaxial needles for serial tumor ablation. Finally, a quick contrast-enhanced CT scan was performed in ETT disconnection and was fused with the planning CT for verification of the ablation size and to exclude possible complications. RF ablation was carried out using the unipolar Cool-tip_RFsystem (Cool-tip, Medtronic, Mansfield, MA, USA), including the Cool-tip_RF switching controller, and a 17G Cool-tip electrode with a length of 25 cm and exposure length of 3 cm. The standard ablation time for three electrodes (switching control) was 16 min. Needle track ablation was done during repositioning and during final removal of the RF-electrodes to prevent bleeding and potential tumor seeding. Example cases of SRFA in octogenarians are shown in Figures 2, 3.

### Endpoints and Follow-Up

The primary endpoints of the study were the primary and secondary technical efficacy, as well as the local recurrence rate (LR). The secondary endpoints included disease-free (DFS) and overall survival (OS). Follow-up contrast-enhanced CT or MR scans were performed at 1 and 3 months intervals after RF ablation. Two experienced abdominal radiologists evaluated the imaging results (PES and BR). Technical success was defined as sufficiently accurate electrode placement according to predefined plans. The appearance of new tumors within or directly adjacent to the ablation zone or initial tumor was judged to constitute LR. Complete ablation was defined as a circumscribed non-enhancing zone within and or extending beyond the initial tumor borders with a clear margin. The appearance of new tumors distant to the ablation zone and or to initial tumor location was defined as distant tumor recurrence. Primary technical efficacy rate was evaluated per lesion as the absence of residual tumor on 1 month follow-up CT. Secondary efficacy rate included tumors that necessitated re-ablation due to a residual tumor. Major complications were defined according to the Society of Interventional Radiology (SIR) Standards of Practice Committee classification (17) and according to the Clavien-Dindo classification (Grade III+) (18). Patient survival was calculated from the date of initial SRFA to the date of death attributable to malignancy or other causes (i.e., event) or the most recent follow-up visit (i.e., censoring).

### Statistical Analysis

The statistical analysis was performed with the software IBM SPSS version 20 (IBM, Armonk, New York). Data were expressed as total numbers, median, and range. The overall survival and disease-free survival were evaluated using the Kaplan Meier method and compared with the log-rank test. The difference between categorical variables was evaluated with the *X*^2^-test, and the difference between independent continuous variables was evaluated with the independent student *t*-test or Mann-Whitney U Test. A *p* < 0.05 was considered as statistical significance.

The software R (version 3.5.2, R Foundation for Statistical Computing, Vienna, Austria) and the R package MatchIt (1:1 matching with the nearest neighbor) was used for the propensity matching process for control group selection.

## Results

### Patient Characteristics (Table 1)

**Figure 4:**
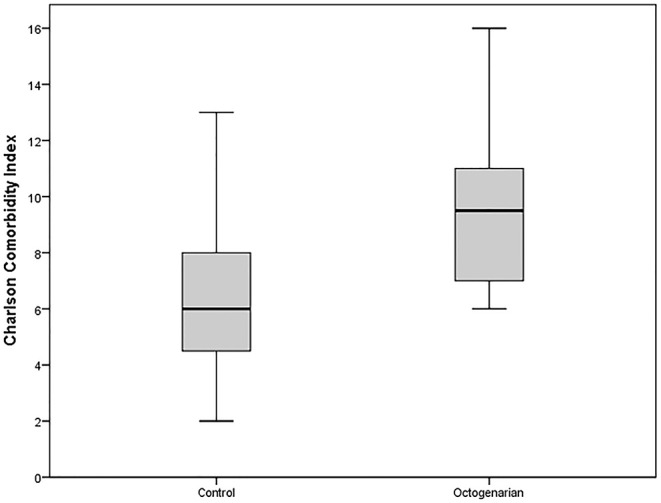
Boxplot presenting Charlson Comorbidity Index of the octogenarian and control group.

Thirty-six patients, 12 females and 24 males, with a median age of 81.5 years (80–89) underwent SRFA for treatment of primary or metastatic liver tumors. 18 (50.0%) were HCCs, 3 (8.3%) intrahepatic cholangiocarcinomas (ICC) and 15 (41.7%) were metastatic tumors of which the majority (60%) were colorectal cancer (CRC) metastases. The median tumor size was 2.7 cm (1.5–9 cm). At first SRFA, 24 patients had a solitary tumor in the liver, 8 had two tumors, 3 had three tumors, and one patient had more than three tumors (multiple nodules). Six patients had undergone previous chemotherapy, six patients surgical resection, four TACE, and two conventional RF ablation (non-stereotactic). The median tumor size was 2.7 cm (1.5–9 cm). Thirty-four (94.4%) patients had concomitant diseases: 25 (69.4%) cardiovascular, 3 (8.3%) respiratory, 17 (47.2%) metabolic, and 7 (19.4%) renal. Twelve (33.3%) had liver cirrhosis affecting the background parenchyma, of which 10 (91.7%) were child A cirrhosis. 6 (16.7) had a secondary tumor. The median charlson comorbidity index (CCI) of the study group was significantly higher vs. the control group, 9.5 (6–16) for the study group and 6 (2–13) for the control group, respectively (*p* = 0.000, Figure 4).

From the control group, 36 patients, 13 females and 23 males, with a median age of 62 years (38–79) underwent SRFA for treatment of 16 (44.4%) HCCs, 3 ICCs (8.3%) and 17 (47.2%) metastatic liver tumors. The median tumor size was 2.5 cm (1.5–11 cm).

### Periprocedural Complications

Major complications post procedure are shown in detail in Table 2. The major complication rate was 10.9% (5 of 46 SRFA sessions). In two patients thermal injury of the adjacent bowel had to be surgically repaired. Other two patients suffered from intra-/perihepatic bleedings that could be treated by angiographic coiling in the same general anesthesia session. A liver abscess in one had to be surgically drained. Using the Clavien-Dindo classification, the complication rate above Grade III was 6.5% (3 of 46 SRFA sessions). There were no periprocedural deaths (30-day).

**Table 2:** Details of major complications after SRFA.

**ID**	**Gr**.	**Age**	**Sex**	**Prim**.	**Cirr**.	**T/S**	**mS/S**	**N/S**	**Complication**	**Therapy**	**SIR**	**CDC**
1	≥80	80	Male	CCC	No	1	2.0 cm	3	Thermal bowel injury	Surgery	Maj.	IIIb
2	≥80	80	Male	HCC	No	1	9.0 cm	11	Liver abscess and thermal injury bowel	Drainage, Surgery	Maj.	IIIb
3	≥80	81	Male	CRC	No	1	5.0 cm	9	Liver abscess	Surgery	Maj.	IIIb
4	≥80	83	Male	HCC	Yes	3	5.8 cm	16	Perihepatic bleeding	AG-coiling	Maj.	–
5	≥80	81	Male	HCC	Yes	2	4.0 cm	10	Intrahepatic bleeding	AG-coiling	Maj.	–
1	<80	45	Female	CCC	No	1	11.0 cm	19	Transient liver failure	ICU	Maj.	IIIB
2	<80	48	Male	HCC	No	1	6.4 cm	12	Transient pulm. failure	ICU	Maj.	IIIB
3	<80	79	Male	CRC	No	1	6.5 cm	9	Liver abscess	US-drainage	Maj.	IIIB
4	<80	57	Male	HCC	Yes	1	9.0 cm	12	Pleural effusion	US-drainage	Maj.	IIIB
5	<80	61	Male	CRC	No	2	4.0 cm	8	Pneumothorax	Chest tube	Maj.	–
6	<80	70	Male	RCC	No	2	6.0 cm	10	Perihepatic bleeding	AG-coiling	Maj.	–
7	<80	48	Male	HCC	No	3	3.0 cm	9	Intrahepatic bleeding	AG-coiling	Maj.	–
8	<80	50	Male	HCC	Yes	3	1.6 cm	7	Perihepatic bleeding	AG-coiling	Maj.	–
9	<80	46	Female	OVC	No	2	3.0 cm	7	Perihepatic bleeding	AG-coiling	Maj.	–

*Gr., group; SRFA, stereotactic radiofrequency ablation; Prim., primary tumor; T/S, tumors per session; mS/S, maximal tumor size per session; N/S, needles per session; HCC, hepatocellular carcinoma; CRC, colorectal carcinoma; RCC, renal cell cancer; OVC, ovarial cancer; Cirr., cirrhosis; AG, angiography; US, ultrasound; SIR, Society of Interventional Radiology; CDC, Clavien-Dindo classification*.

The median post procedure hospital stay was 6 days, ranging from 2-33 days for the octogenarian group and 4 days, ranging from 1 to 33 days for the control group, respectively (p = 0.370).

In the control group, the major complication rate was 13.4% (9 of 67 SRFA sessions), including transient liver-/pulmonary failure (two patients), intra-/perihepatic bleedings (four patients), pneumothorax (one patient), and pleural effusions (two patients). There was no significant difference compared with the octogenarian group (*p* = 0.685).

### Technical Success

SRFA was completed according to predefined plan in all 70 tumors (technical success rate 100%). 68/70 tumors were successfully ablated at initial SRFA (97% primary efficacy rate). Both residual tumors were successfully retreated, resulting in a secondary efficacy rate of 100%. 1–15 (median four) RF electrodes were inserted in each tumor.

Compared to the control group, there was no significant difference of primary or secondary efficacy (94.7%, 107/113, *p* = 0.292; 99.1%, 112/113, *p* = 0.430). There was also no significant difference based on tumor entity (*p* = 0.285 and *p* = 0.548 for HCC and *p* = 0.464 and *p* = N/A for CRC patients, respectively). Detailed description is shown in Tables 3, 4.

**Table 3:** Local tumor control after SRFA.

**ID**	**Age**	**Sex**	**Prim**.	**Cirr**.	**Size**	**Needles**	**Seg**.	**Properties**	**Ablation time**	**Pre-therapy**	**Outcome**
1	86	Female	CRC	Yes	1.5 cm	3	VII	Subcaspular	16 min	CTX	LR
2	85	Male	CRC	Yes	1.5 cm	3	VIII	–	16 min	CTX	LR
3	80	Female	NET	Yes	2.5 cm	5	III	Gallbladder	26 min	HR	LR
4	80	Male	HCC	Yes	6.9 cm	7	IV	–	61 min	–	LR
4	80	Male	HCC	Yes	3.8 cm	3	III	Subcapsular	61 min	–	LR
5	87	Male	CRC	No	6.5 cm	11	VI	Subcapsular	90 min	–	IA
6	81	Male	CRC	No	4.0 cm	6	VII	Organ	32 min	–	IA

*SRFA, stereotactic radiofrequency ablation; Prim., primary tumor; HCC, hepatocellular carcinoma; CRC, colorectal carcinoma; NET, neuroendrocrine tumor; Cirr., cirrhosis; Seg., liver segment; HR, hepatic resection; IA, incomplete ablation; LR, local recurrence*.

**Table 4:** Tumor based therapy success rates compared to control group.

**Rate**	**Octogenarian Gr**.	**Control Gr**.	***P*-value**
Technical success, *n* (%)	70/70 (100)	113/113 (100)	N/A
Primary technical efficacy, *n* (%)	68/70 (97.1)	107/113 (94.7)	0.292
HCC, *n* (%)	38/38 (100)	44/46 (95.7)	0.285
CRC, *n* (%)	17/19 (89.5)	23/24 (95.8)	0.464
Secondary technical efficacy, *n* (%)	70/70 (100)	112/113 (99.1)	0.430
HCC, *n* (%)	38/38 (100)	45/46 (97.8)	0.548
CRC, *n* (%)	19/19 (100)	24/24 (100)	N/A
Local recurrence, *n* (%)	5/70 (7.1)	12/113 (10.6)	0.431
HCC, *n* (%)	2/38 (5.3)	7/46 (15.2)	0.132
CRC, *n* (%)	2/19 (10.5)	4/24 (16.7)	0.505

*HCC, hepatocellular carcinoma; CRC, colorectal carcinoma; Gr., group; N/A, not available*.

### Local Recurrence Rate and Distant Recurrence

Local tumor recurrence developed in 5 of 70 nodules (7.1%, detailed description in Table 4), distant tumor recurrence in the liver was found in 14 patients (38.9%). Of the 14 patients who experienced intrahepatic recurrences, 9 (25%) patients received repeated SRFA. Following this, seven patients (19.4%) developed untreatable tumor progression. The mean imaging follow-up was 15.6 months (0.3–90.6).

The local recurrence rate of the control group was 10.6% (12/113 tumors). There was no significant difference (*p* = 0.431). A sub analysis based on tumor type (HCC and CRC) did also show no significant differences (*p* = 0.132 and *p* = 0.505, respectively).

### Overall and Disease-Free Survival (Figure 5)

In the octogenarian group, the overall survival rates at 1-, 3-, and 5- years from the date of the first SRFA were 84.6, 50.5, and 37.9% for HCC patients and 87.5, 52.5, and N.A.% for CRC patients, with a median overall survival (OS) of 51.5 months (CI 19.2-n.a.) and 51.4 months (CI 19.6-n.a.), respectively. The disease-free survival (DFS) for HCC patients after SRFA was 79.1, 35.6, and 23.7%, at 1-, 3-, and 5-years, respectively, with a median DFS of 19.2 months and 75, 22.5, and N.A.%, with a median DFS of 16.2 months for CRC patients, respectively.

**Figure 5:**
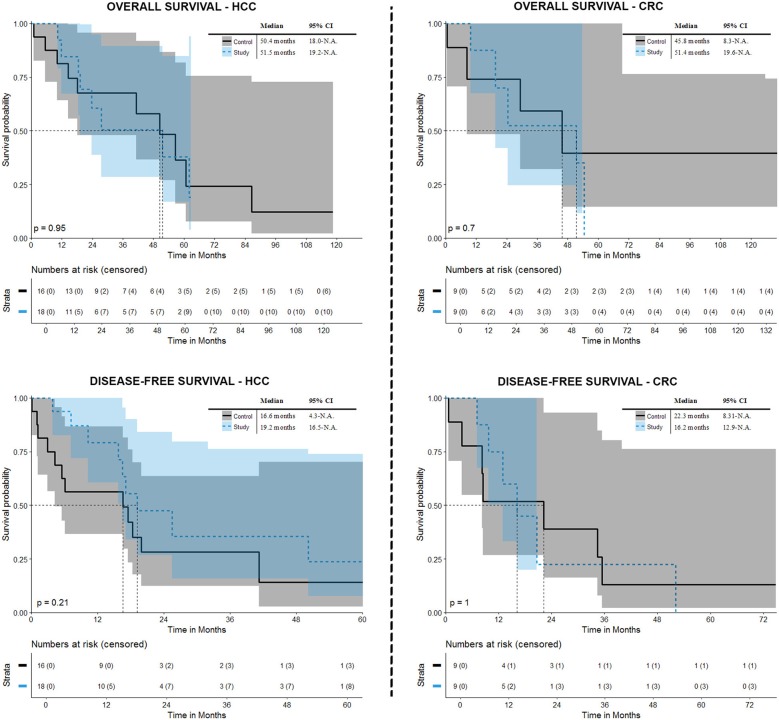
Overall and disease-free survival after initial SRFA.

In terms of OS, there was no significant difference between control and octogenarian group, *p* = 0.952 for HCC (median 50.4 months) and *p* = 0.704 for CRC patients (median OS 45.8 months), respectively. Regarding DFS there was also no significant difference compared to the control group (*p* = 0.214 with a median survival 16.6 months for patients suffering from HCC and *p* = 0.999 with a median survival 22.3 for patients suffering from CRC).

## Discussion

This study has shown that SRFA is a feasible option for treating liver tumors in octogenarians. This is of central importance in this cohort of patients who also have additional risk factors due to age related co-morbidity. In the present study, this cohort of patients had a significantly higher Charlson Comorbidity Index compared with the younger control cohort (median 9.5 vs. 6, *p* = 0.000). The median length of hospital stay was 6 days (range 2–33 days) in the patients from the octogenarian group and 4 days in the control group. These relatively long hospital stays in both groups—as compared to conventional ultrasound- and CT- guided ablation techniques—may be explained by the necessity of general anesthesia for reliable needle placement and the large tumor sizes with a median tumor size of 2.7 cm and a maximum tumor size of 9 cm. The longer hospital stays in the octogenarian group may be explained by age related co-morbidities, availability of care at home and discharge related issues. The in the literature reported median hospital stays after hepatic resection (HR) in older patients range from 11 to 12 days and is substantially longer (19–21). This however, has been reported to be a median of 6 days for octogenarians undergoing HR in programs using enhanced recovery after surgery (22). The importance of the length of hospitalization is emphasized by the fact, that older patients show a significant decline of functional status which is directly related to the length of hospitalization (23).

According to the SIR guidelines the major complication rate in our patient series was 10.9%, notably no difference to the control group (*p* = 0.685), including two thermal injuries of the bowel and one liver abscess that all had to be surgically treated. Two major bleedings could be treated with interventional radiology at the index procedure. Using the Clavien-Dindo classification the major (Grade 3+) complication rate was 6.5%. In contrast, Riffat et al. (24) reported a major (Clavien-Dindo 3+) complication rate of 27% after HR in octogenarians. In line with this Bhandari et al. (25), and Makridis et al. (21) quoted perioperative morbidity of 37 and 34.8% after HR in octogenarians, respectively. Takahashi et al. (26) reported a major complication rate of 2.8% after conventional RF ablation of HCCs up to 5 cm in older (≥75 years) patients. Compared to our patient cohort, these patients were younger, the treated tumors smaller and the number of used RF probes smaller (1 probe in tumors up to 2 cm, vs. median 4 RF probes). Moreover, when looking at the ablations where major complications occurred in our series, the median number of inserted needles was 10. Similar to Takahashi et al. (26), there was no perioperative death within 30 days after SRFA in our cohort. In comparison Riffat et al. (24), Adam et al. (27), and Reddy et al. (28) reported mortality rates of 6.7, 3.8, and 6.2% after HR, respectively.

In the present study, the primary technical efficacy, i.e., complete ablation after SRFA was 97.1%. Our group recently (29), reported complete pathological response (i.e., no evidence of tumor) in 97.3% in a histopathological study in explanted livers where SRFA was used as a bridging procedure to liver transplantation. These results were not significantly different in our non-octogenarian matched control group (94.7%, *p* = 0.292). When considering technical success of ablation of liver tumors in older patients however the literature is sparse. In small HCC (<3 cm), the reported complete response rates after RF ablation exceed 90% (30, 31). However, these rates significantly decrease (40–70%) in the treatment of larger HCC nodules (32, 33). In line with this Lin et al. (34) reported primary efficacy rates of 79% in medium sized (3.1–5 cm) HCC tumors after US-guided RF ablation in a younger population. However, more recent studies show better results with primary efficacy rates up to 91–97% owing to the usage of multi-probe RF ablation in medium and large HCCs (34, 35) in younger patients. Use of the stereotactic technique with the application of multiple internally cooled RF probes paired with consistent, exact three-dimensional probe alignment creating sufficiently overlapping coagulation zones makes the treatment of even large tumors technically feasible. In accordance Widmann et al. (36) showed a primary efficacy rate of 95.5% after a single SRFA in an average patient series. In our study, local tumor recurrence developed in 7.1% (5/70) in the octogenarian group with no significant difference to the control group (*p* = 0.431). This result is slightly superior to the results of Takahashi et al. (26) who reported a local recurrence rate of 10.1% after RF ablation of HCC in older patients and to the results of Yazici et al. (37) with a local recurrence rate of 29% after laparoscopic RF ablation of malignant liver tumors in older patients (≥65). However, the same authors showed a very low local recurrence rate of 2.4% for laparoscopic resection in the same study.

Among the octogenarians in the present study the patients suffering from HCC showed a median OS of 51.5 months with 1-, 3-, and 4-years OS rates of 84.6, 50.5, and 37.9%. These results were not significantly different to the control group. The reported OS after HR are comparable: Ferrero et al. (38), Oishi et al. (39), and Kondo et al. (40) showed a 5-years OS in older HCC patients of 48.6, 58, and 43%, respectively. Furthermore, Gupta et al. (41) reported a 5 years OS of 47% after HR of primary and secondary liver tumors. Takahashi et al. (26) reported OS rates of 82% at 3 years and 61% at 5 years after RF ablation in HCC patients in the older patients group (≥75). These results were not significantly different from the younger group in their study. In line with this, Tateishi et al. (42) reported a 3-years OS of 76% after RF ablation of HCCs in in a younger cohort of older patients (≥68 years) with no difference to the younger patients in the study. In terms of CRLM, the 3-years OS was 37.9% in our octogenarian group. Admittedly, due to the small patient number, the 5-years OS in CRLM patients was not calculable with the Kaplan-Meier method. However, these results are comparable to Tufo et al. (22) and Nardo et al. (43), who showed OS rates of 79.4, 37.3, and 20.4% and 85.7, 38.9 and 28.6% at 1-, 3-, and 5-years, respectively, after HR of CRLM in octogenarians.

In our study, the 5-years DFS for HCC patients was 23.7%, with a median DFS of 19.2 months, and the 3-years DFS was 22.5% in CRLM patients, with a median DFS of 16.2 months. Kaibori et al. (14) reported a 5-years DFS of 33.2% and 23.2% after HR and RFA in older HCC patients, respectively, being significantly higher after HR. Gupta et al. (41) showed a 5-years DFS of 37% after HR of malignant liver tumors in octogenarians. Tufo et al. (22) showed a DFS at 3 and 5 years of 31.5% and 16.6% after HR of CRLM in octogenarians. The discrepancy between OS and DFS in our study and comparison to the given results after HR could be due to the fact that most patients were retreated on recurrences and thus resulting in a minor impact on OS.

### Limitations

The limitations of this study are related to its retrospective design and the small number of patients. Especially, when looking at the survival plots, the CI-intervals are large and thus could diminish the validity of the expected survival rates. However, propensity score matching allowed for adequate selection of patients for the control group. Another limitation is the possible bias of additional therapies after SRFA, such as TACE or CTX on the overall clinical outcome that could not be fully eliminated.

In conclusion, regardless of the limitations mentioned above, SRFA in octogenarians is a safe, feasible and useful option in the management of primary or metastatic liver tumors with no significant differences in terms of technical efficacy, local recurrences, major complications, OS and DFS compared to the younger control group cohort. Considering the increasing likelihood of adverse effects of hospitalization with increasing duration of stay, especially in older patients, with a decline in functional status or increased risk of psychotic events, it is in particular important to older patients to minimize the therapeutic aggression and thus shorten the hospitalization. The present study suggests that SRFA as a minimally invasive treatment option could achieve this goal.

## Data Availability

The datasets generated for this study are available on request to the corresponding author.

## Ethics Statement

The studies involving human participants were reviewed and approved by Institutional Review Board Medical University of Innsbruck. The patients/participants provided their written informed consent to participate in this study.

## Author Contributions

PS contributed to study concepts, study design, data acquisition, data analysis, statistical analysis, and manuscript preparation and editing. DP and GL contributed to data acquisition and manuscript review. MS and CK contributed to manuscript editing and review. RB contributed to study concepts, study design, data analysis, and manuscript review.

### Conflict of Interest Statement

RB is a consultant for CAScination and i-Sys Medizintechnik. The remaining authors declare that the research was conducted in the absence of any commercial or financial relationships that could be construed as a potential conflict of interest. The reviewer RR declared a shared affiliation, with no collaboration, with the author MS to the handling editor at the time of review.

## Abbreviations

DFS: disease-free survival
HCC: hepatocellular carcinoma
HR: hepatic resection
ICC: intrahepatic cholangiocarcinoma
IN: incomplete necrosis
LR: local recurrence
OS: overall survival
RF: radiofrequency
SRFA: stereotactic radiofrequency ablation
TACE: transcatheter arterial chemoembolization.
